# NOG-Derived Peptides Can Restore Neuritogenesis on a CRASH Syndrome Cell Model

**DOI:** 10.3390/biomedicines10010102

**Published:** 2022-01-04

**Authors:** Matteo Gasparotto, Yuriko Suemi Hernandez Gomez, Daniele Peterle, Alessandro Grinzato, Federica Zen, Giulia Pontarollo, Laura Acquasaliente, Giorgia Scapin, Elisabetta Bergantino, Vincenzo De Filippis, Francesco Filippini

**Affiliations:** 1 Synthetic Biology and Biotechnology Unit, Department of Biology, University of Padua, 35131 Padua, Italy; matteo.gasparotto.1@phd.unipd.it (M.G.); yurikosuemi.hernandezgomez@gmail.com (Y.S.H.G.); alessandro.grinzato@phd.unipd.it (A.G.); federica.zen.20@gmail.com (F.Z.); elisabetta.bergantino@unipd.it (E.B.); 2 Department of Pharmaceutical and Pharmacological Sciences, University of Padua, 35131 Padua, Italy; d.peterle90@gmail.com (D.P.); giulia.pontarollo@studenti.unipd.it (G.P.); laura.acquasaliente@unipd.it (L.A.); 3 Department of Medicine, Harvard Medical School, Boston, MA 02115, USA

**Keywords:** neuritogenesis, NOG motif, L1CAM, homophilic binding, biomimetic peptide, CRASH syndrome, neurodevelopmental disorder, regenerative medicine, neuronal differentiation

## Abstract

Homo- and heterophilic binding mediated by the immunoglobulin (Ig)-like repeats of cell adhesion molecules play a pivotal role in cell-cell and cell-extracellular matrix interactions. L1CAM is crucial to neuronal differentiation, in both mature and developing nervous systems, and several studies suggest that its functional interactions are mainly mediated by Ig2–Ig2 binding. X-linked mutations in the human *L1CAM* gene are summarized as L1 diseases, including the most diagnosed CRASH neurodevelopmental syndrome. In silico simulations provided a molecular rationale for CRASH phenotypes resulting from mutations I179S and R184Q in the homophilic binding region of Ig2. A synthetic peptide reproducing such region could both mimic the neuritogenic capacity of L1CAM and rescue neuritogenesis in a cellular model of the CRASH syndrome, where the full L1CAM ectodomain proved ineffective. Presented functional evidence opens the route to the use of L1CAM-derived peptides as biotechnological and therapeutic tools.

## 1. Introduction

During development, neurons extend their axons to find the right targets in a complex and changing environment and establish a functional synaptic network. Each axon is tipped with the growth cone, a specialized structure with highly dynamic behavior and responsiveness to multiple sources of spatial information, hence guiding the axon itself toward the right targets with an impressive level of accuracy [1]. Cell adhesion molecules (CAMs) with immunoglobulin (Ig)-like repeats play a fundamental role in cell-cell and cell-extracellular matrix (ECM) interactions in both mature and developing nervous systems, as well as in axonal regeneration and neural repair [2]. These proteins are prototyped by L1CAM, which exhibits a large extracellular domain (ED) consisting of six Ig-like repeats, followed by five fibronectin type III regions [3].

L1CAM plays an important role in neuronal differentiation: it can induce neuritogenesis, guide axonal growth cones through laminin-induced haptotaxis [4], fasciculation, and is involved in cell migration and survival [5]. L1CAM homophilic trans binding is responsible for triggering neurite outgrowth. Structural and functional studies determined that this interaction in L1 family proteins is mediated by the first four Ig-like domains arranged in a horseshoe conformation, where Ig1 and Ig2 fold back to interact with Ig4 and Ig3, respectively [6]. Cryoelectron tomography of L1CAM suggested that homophilic binding could occur between edges of Ig2 domains, with a perpendicular association between two horseshoe structures or between the large faces of two Ig2–3 dimers [7]. Surface plasmon resonance was used to define the kinetic details of this interaction, and the analysis confirmed that the horseshoe (Ig1–Ig4) is the main region required for homophilic binding [8].

The pivotal role of L1CAM in driving neuritogenesis results in a strong involvement in neurological disorders. Mutations in the X-linked, human *L1CAM* gene are summarized as L1 diseases, including MASA syndrome (intellectual disability, aphasia, shuffling gait, adducted thumbs), X-linked hydrocephalus (XLH) due to stenosis of the aqueduct of Sylvius (HSAS), X-linked agenesis of corpus callosum (ACC), and spastic paraparesis type 1 (SP1) [9]. However, the most commonly diagnosed is the CRASH syndrome, which is characterized by corpus callosum hypoplasia, mental retardation, adducted thumbs, spastic paraplegia, and hydrocephalus [10]. Such X-linked disorders mostly affects males, with an incidence of 1:30,000 newborn [11]. According to “The L1CAM Mutation Database” [11], Ig2 harbors the highest number of known disease-causing mutations, suggesting hindering L1CAM homophilic binding plays the main role in CRASH syndrome etiology. Indeed, mutations M172I and D202Y impair homophilic interaction at the cell surface [12]; in the same binding region, mutations I179S, R184Q [13] and Y194C [14] are also associated with HSAS and MASA. The R184Q mutation was extensively described by Jouet and coworkers, and associated with hydrocephalus, CNS deformity, mental retardation, speech problems, limb spasticity, adducted thumbs, and a poor prognosis (10 out of 15 patients died before age 1) [15]. This mutation has been found to strongly reduce homophilic, heterophilic binding and neurite outgrowth [16,17]. Conversely, the I179S mutation results in relatively milder symptoms, including adducted thumbs, mental retardation, and spastic paraplegia [18,19], and causes a reduced stimulation of neurite outgrowth from mouse cerebellar neurons grown onto a fibroblast feeder layer [20]. It is noteworthy that L1-A, a 14 aa peptide derived from the same homophilic binding region of L1CAM Ig2 (residues 178–191) [17], proved able to trigger L1CAM effects on cell morphology [21].

This region has recently been found to be part of a Neurite Outgrowth and Guidance (NOG) motif, which is 100% conserved in mammals and man among roughly ten thousand of neuronal CAMs, including all four members of the L1 family proteins (L1CAM, Close Homolog of L1 or CHL1, NrCAM, and Neurofascin), Contactins, Deleted in Colon Carcinoma (DCC)/Netrin receptor and Roundabout (ROBO) receptors [22]. In such proteins, the NOG region is part of a beta-hairpin loop stabilized by a salt bridge between a fully conserved arginine and aspartic acid residue. Synthetic peptides derived from the NOG motifs of these CAMs are unstructured and stable for at least 48 h in culture media. Most importantly, they all share proneuritogenic capacity and have been found to bind L1CAM ectodomain in competition surface plasmon resonance assays [22]. 

Several CRASH-causing mutations fall into the NOG region of L1CAM, and interestingly the R184Q mutation hampers the fully conserved arginine of the NOG motif [22]. This prompted us to set up a model system to evaluate the effect of such mutations on neuritogenesis and to exploit the potential of biomimetic peptides as either negative or positive regulators. To this aim, we used two different, NOG-derived peptides: L1-A, as it is derived from L1CAM, and ROBO2-A, as derived from a CAM outside the L1CAM family. We performed in vitro and in silico analyses to gain insights on the mechanisms mediating the L1CAM homophilic binding, how such binding is hampered by the CRASH mutations, and how to rescue the neuritogenesis in neuronal cultures bearing the CRASH mutations. We report here that severity of CRASH phenotypes caused by I179S and R184Q mutations matches the residual proneuritogenic capacities in vitro of the corresponding L1CAM mutant proteins and peptides. In silico simulations provided a molecular rationale for Ig2 impairment and drove design of rescue assays, which resulted in promising evidence. NOG-derived peptides and L1CAM ED have comparable potency in triggering neurite outgrowth, but only peptides can also rescue the neuritogenesis in a CRASH syndrome cellular model. Together, our results open the route to the use of L1CAM-derived peptides as both biotechnological and therapeutic tools.

## 2. Materials and Methods

### 2.1. Structural Modeling and Molecular Dynamics

Models of wild type L1CAM and its mutants were obtained through homology modeling via SWISS-MODEL [23,24,25] using the neurofascin dimer crystal structure as template (PDB 3P3Y). Original models were refined using SCWRL [26,27] and model quality was assessed via QMEAN server [28].

Molecular dynamics (MD) was performed with Gromacs 2016.1 [29], using the Amber99 force field [30], in order to investigate the role of mutations on the structural organization of the L1CAM protein. The models were solvated with the TIP3P water model in a rectangular box with a minimum distance of 1 nm between the protein and the border. NaCl 0.15 M was added to simulate a physiological ionic strength.

After the energy minimization, the temperature was set to 310 K and equilibrated for 100 ps with the Berendsen thermostat [31]. Similarly, the pressure was equilibrated to 0.01 atm for 10 ns using the Parrinello-Rahman barostat [32]. The production simulation was performed for 300 ns, until the model reached stable conformation, as confirmed by RMSD. Electrostatic and van der Waals interactions were calculated using the particle mesh Ewald potential [33] with a 1 nm cut-off. The displacement of the four domains was determined by calculating the distance between Cα atoms of the L1CAM-wt and the mutated models.

Final structures were compared using UCSF Chimera [34,35] v. 1.8.1 and binding pockets were determined by submitting structures to the PeptiMap webserver [36,37]. All atom RMSD calculation on final structures was performed with PyMol [38] align algorithm, setting iteration cycles to zero.

### 2.2. Peptide Synthesis, Purification and Characterization

All peptides were synthesized in our laboratory by the solid-phase method using the flurenylmethyloxycarbonyl (Fmoc) chemistry [39,40] on a model PS3 automated synthesizer (Protein Technologies International, Tucson, AZ, USA). The peptides were assembled stepwise on a Wang resin (Novabiochem) derivatized with the desired corresponding C-terminal amino acid. Removal of N^a^-Fmoc-protecting groups was achieved by treatment for 20 min at room temperature with a deprotection solution (20% piperidine in N-methylpirrolidone—NMP). Standard coupling reactions were performed with an equal molar ratio of 2-(1H-benzotriazol-1-yl)-1,1,3,3-tetramethyluronium hexafluorophosphate and 1H-hydroxy-benzotriazole as activating agents, with a four-fold molar excess of N^α^-Fmoc-protected amino acids in activation solution. For double coupling at peptide bonds involving Val, Ile, Leu, and Phe, the stronger activator 2-(7-aza-1H-benzotriazol-1-yl)-1,1,3,3-tetramethyluronium hexafluorophosphate was used. Once the peptide assembly was completed, the side chain-protected peptidyl resin was treated for 90 min at room temperature with the following mixture: 92.5% TFA, 2.5% H_2_O, 2.5% ethandithiol, and 2.5% triisopropylsilane. The resin was removed by filtration, and the acidic solution, containing the unprotected peptide, was precipitated with ice-cold tertbutyl-methylether and then lyophilized.

Peptides were purified to homogeneity (>98%) by RP-HPLC (HPLC Pu-1575 equipped with 1575 UV-Vis detector; Jasco, Tokyo, Japan) on a semipreparative Vydac (Grace, Hesperia, CA, USA) C18 column (10 × 250 mm, 5 µm particle size, 300 Å porosity) equilibrated with 0.1% (*v*/*v*) aqueous TFA and eluted with a linear 0.078% (*w*/*w*) TFA-acetonitrile gradient at a flow rate of 1.5 mL/min. Absorbance of the effluent was monitored at 226 nm [35]. Purified peptides were analyzed using a Xevo-G2S Q-TOF instrument (Waters, Milford, MA, USA), which yielded mass values in agreement with the theoretical mass within 2 ppm accuracy. Concentration of peptides with aromatic amino acids was determined on a V-630 spectrophotometer (Jasco, Tokyo, Japan) by measuring the absorbance at 257 nm for Phe-containing peptides, or at 280 nm for Tyr-containing peptides, using a molar absorptivity of 200 M^−1^∙cm^−1^ or 1280 M^−1^∙cm^−1^ for Phe or Tyr, respectively. For peptides lacking any suitable chromophore, the concentration was determined by analytic scaling (E/50, Gibertini Elettronica, Novate Milanese, MI, Italy).

### 2.3. Cell Culture and Peptide Treatment

Exponential growing human neuroblastoma cell line SH-SY5Y [41] was cultured with Dulbecco’s Modified Eagle Medium/Nutrient Mixture F-12 with GlutaMAX™ supplement (DMEM/F-12, Invitrogen Life Technologies, San Giuliano Milanese, MI, Italy) supplemented with 10% heat-inactivated fetal bovine serum (FBS, Euroclone) and 25 μg/mL of gentamicin (Sigma Aldrich, Milano, MI, Italy) (growth medium), in a humidified atmosphere of 5% of CO_2_ in air at 37 °C. Cultures were maintained by subculturing cells into 25 cm² flasks (Sarstedt AG & Co. KG, Nümbrecht, Germany) once they reached roughly 80% confluence. In experiments with peptides added to the culture medium, cells were seeded in a 24-well plate (25,000 cells/well) coated with a gelatin (Sigma Aldrich, MI, Italy)/poly-L-lysine (Invitrogen, MI, Italy) solution. Poly-L-lysine is widely used as a good substrate for neural cell adhesion and growth. At 24 h after cell seeding (day 0), the growth medium was replaced by medium supplemented with peptides, except for control samples.

### 2.4. L1CAM Clones and SH-SY5Y Transfection

A cDNA including the wild type coding sequence (CDS) of human, neuronal L1CAM was received as a kind gift from Prof. Jacopo Meldolesi’s lab (University of Milan, Milan, MI, Italy). Then, some CDS regions were modified by synthetic DNA (from GeneArt^(TM)^ service, Life Technologies, MI, Italy) in order to (i) delete and add restriction sites (silent mutations, not altering the final amino acid sequence), (ii) create an L1CAM-EGFP fusion protein (C-terminal EGFP tag), and (iii) to obtain mutant L1CAM harboring R184A and R184Q mutations. For transient expression, each CDS was cloned into a modified version of plasmid pEGFP-N1 (Takara Bio Europe SAS, Saint-Germain-en-Laye, France), and they were used in transfection experiments. SH-SY5Y cells in proliferating conditions were transfected using Lipofectamine 2000 (Invitrogen, MI, Italy) following manufacturer protocol. In particular, 2.5 × 10^4^ cells were transfected with 0.35 µg DNA and 0.5 µL Lipofectamine, using the plasmid expressing EGFP alone as control. Transfection media was replaced by culture medium 6 h after transfection. In experiments combining transfections and peptide treatment, peptides were added 6 h after transfection and cells were observed 24 h after peptide treatment (30 h after transfection).

### 2.5. Neuritogenesis Assays

Neurite outgrowth was measured after staining cells with Calcein-AM (Biotium, Fremont, CA, USA; 2 µM in HBSS, Hank’s Balanced Salt Solution, Invitrogen, MI, Italy) and Hoechst 33258 (Invitrogen Life Technologies, MI, Italy 10 µg/mL) for 30 min in the dark at 37 °C and 5% CO_2_. Cells were then washed, and medium was replaced with fresh HBSS. Cells were observed with a DMI4000 microscope (Leica Microsystem GMbH, Wetzlar, Germany) with a 10× magnification using a GFP and DAPI filter. Ten images/well were recorded; the first two fields were set to correspond to the center of the well. Next, fields were then selected in the periphery of the well (N, NE, E, SE, S, SW, W, NW, with respect to the center), so that images could be representative of the whole well. Images were evaluated with Fiji [42]. Cell numbers were inferred by manually counting nuclei. Neurite length was measured by tracing the trajectory of the neurite from the tip to the junction between the neurite and cell body. If a neurite exhibited branching, the measure from the end of the longest branch to the soma was recorded, then each branch was measured from the tip of the neurite to the neurite branch point.

Only neurites longer than 50 µm were considered [43,44], and, in experiments with transfection, only transfected cells were analyzed. The neuritogenic properties were analyzed in terms of total neurite length/no. of cells (aggregate length of all cellular processes divided by cell number) and no. of neurites/no. of cells. Values were then normalized to the untreated proliferative control and reported in percentage. Each experiment was performed in three independent replicates.

The EC_50_ values of the dose-response curves were obtained as a fitting parameter by plotting the response of the cell culture at different peptide concentration. Data analysis was performed by GraphPad Prism v8, using the following equation, describing the three parameters (agonist) vs. response model:

(1) $$f\left(x\right)=\mathrm{Min}+\frac{x\cdot \left(\mathrm{Max}-\mathrm{Min}\right)}{{\mathrm{EC}}_{50}+x}$$

Similarly, IC_50_ values were obtained by interpolating experimental points with the following equation, describing the three parameter (inhibitor) vs. response model:

(2) $$f\left(x\right)=\mathrm{Min}+\frac{\mathrm{Max}-\mathrm{Min}}{1+\left(x/{\mathrm{IC}}_{50}\right)}$$

In both cases, Min and Max values are the plateau values and x the concentration of the agonist or antagonist.

### 2.6. Immunofluorescence

Cells were transfected with L1CAM mutants and fixed in 4% paraformaldehyde (PFA) for 15 min. Then, samples were blocked in 0.5% BSA in PBS for 45 min at room temperature (RT). Staining was performed for 90 min at RT using anti-L1CAM primary antibody (Santa Cruz Biotechnology, Inc., Heidelberg, Germany) diluted in 3% BSA in PBS. Secondary antibody (Alexa Fluor 544, ThermoFisher, Rodano, MI, Italy) was diluted in 0.5% BSA and incubated for 45 min at RT. Finally, coverslips were mounted with mowiol mounting medium. After 24 h of polymerization, samples were observed using a Leica SP5 confocal microscope.

### 2.7. Statistical Analysis

Statistical analysis was performed using GraphPad Prism v8. Unless otherwise specified, unpaired, two-tailed Student’s t-test was performed, and results were considered significant when *p* < 0.05.

## 3. Results and Discussion

### 3.1. NOG-Derived Peptides and L1CAM ED Show Comparable Proneuritogenic Capacity

**Figure 1:**
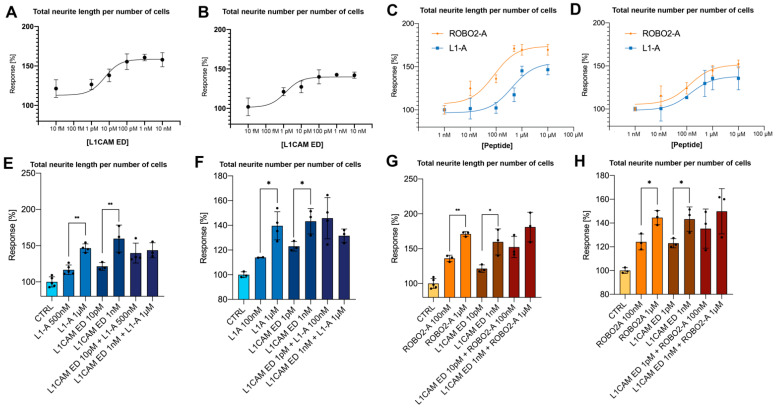
Effects of L1CAM ED and NOG-derived peptides on neuritogenesis. (**A**) Total neurite length per number of cells and (**B**) neurite number per number of cells of proliferating SH-SY5Y cells treated with increasing concentration of L1CAM ED (from 10 fM to 10 nM). (**C**) Total neurite length per number of cells and (**D**) neurite number per number of cells of proliferating SH-SY5Y cells treated with increasing concentration (from 1 nM to 10 µM) of either L1-A (blue) or ROBO2-A (orange). Total neurite length per number of proliferating SH-SY5Y cells treated with combination of L1-A (**E**) or ROBO2-A (**G**). Neurite number per number of proliferating SH-SY5Y cells treated with combination of L1-A (**F**) or ROBO2-A (**H**). All data represent the mean ± SD of at least three independent experiments. Significance at *p* < 0.05 (*), *p* < 0.001 (**), between treated samples and untreated control is reported.

L1-A derives from the NOG motif of L1CAM, and it is suggested to mimic the homophilic binding of its extracellular domain (L1CAM ED) [17]. Moreover, all NOG-derived peptides have recently proven to enhance neuritogenesis in cell culture, possibly by binding to the ectodomains of a number of neuronal CAMs [22]. As peptides and L1CAM ED can trigger the same process, we wondered if some difference could be observed between them in terms of neuritogenic efficacy. Figure 1A,B show the neuritogenic effect of L1CAM ED on proliferating SH-SY5Y cells. Specifically, neuritogenic activity starts from the picomolar range, and reaches plateau around 1 nM, i.e., with an estimated EC_50_ = 8.65 pM for neurite elongation and EC_50_ = 1.07 pM for neurite sprouting. Such curves are similar to those of L1-A and ROBO2-A (representative for peptides derived from NOG-positive CAMs other than L1 family ones) reported in Figure 1C,D: both peptides are active in the high-nanomolar range and reach plateau at 1 µM. Both peptides are similar in promoting neurite sprouting (ROBO2-A EC_50_ = 132 nM, L1-A EC_50_ = 140 nM), however, ROBO2-A seems to be slightly more efficient in promoting neurite elongation (ROBO2-A EC_50_ = 84 nM, L1-A EC_50_ = 407 nM).

When comparing protein and peptide effects, L1CAM ED is effective at 1000-fold lower concentration than peptides in promoting both processes. This is not unexpected, as often whole proteins or their domains are active at lower concentrations than mimetic motifs or drugs. However, both molecules display the same neuritogenic efficacy, with maximum effect around +160% in terms of increased neurite length and +140% in terms of increased neurite number. Then, cells were treated with combinations of NOG-derived peptides and L1CAM ED at their optimal or EC_50_ concentration (Figure 1E–H). When L1CAM-driven increase in neuritogenesis is at its maximum level (e.g., when treating cells with 1 nM L1CAM ED), no further increase can be obtained by exogenous addition of NOG-derived peptides even if they are administered at the optimal concentration of 1 µM, and vice versa. The addition of both molecules at their semi-maximal concentration resulted in an additive effect. However, their combined effect was comparable to that of either peptides or the protein at their optimal concentration.

### 3.2. L1-A Peptides with Known L1CAM Mutations Show Impaired Neuritogenic Capacity

**Figure 2:**
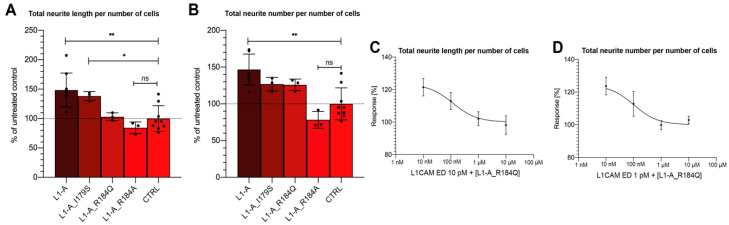
Effect of CRASH-derived peptides on proliferating SH-SY5Y cells. (**A**) Total neurite length per number of cells, (**B**) neurites per number of cells. Treatment with mutant peptides hampers neurite elongation, whereas has a limited effect on sprouting. (**C**) Total neurite length per number of cells treated with 10pM L1CAM ED and increasing concentration of L1-A_R184Q (from 10nM to 10 µM). (**D**) Total neurite length per number of cells treated with 1pM L1CAM ED and increasing concentration of L1-A_R184Q (from 10 nM to 10 µM). All data represent the mean ± SD of at least three independent experiments. Significance at *p* ≥ 0.05 (ns), *p* < 0.05 (*), *p* < 0.001 (**), between treated samples and untreated control is reported.

NOG-derived peptides can promote neuritogenesis by partially reproducing the NOG-motif. Thus, we wondered if some of its known mutations would also reproduce their effects in corresponding L1-A mutant peptides. As Zhao and coworkers mutated Arg-184 to Ala and found that binding capacity of L1-A_R184A was suppressed [17], this nonnatural mutation was also tested for comparison. Therefore, we synthesized the following peptides: L1-A_R184Q, L1-A_I179S (reproducing CRASH mutations), and L1-A_R184A, which were used in a new set of neuritogenesis assays. Figure 2A,B show that the effect of the two CRASH mutations is confirmed even at the peptide level, as both peptides mutated at Arg-184 lose neuritogenic capacity, whereas I179S mutation only partially impaired L1-A biomimetic capacity.

We also wondered if these nonneuritogenic peptides could interfere with L1CAM ED-mediated neuritogenesis. When L1-A_R184Q was used in competition experiments with L1CAM ED, it was able to block its proneuritogenic activity at concentrations as low as 100 nM (Figure 2C,D).

### 3.3. CRASH Mutations Differentially Impact on L1CAM Proneuritogenic Activity

**Figure 3:**
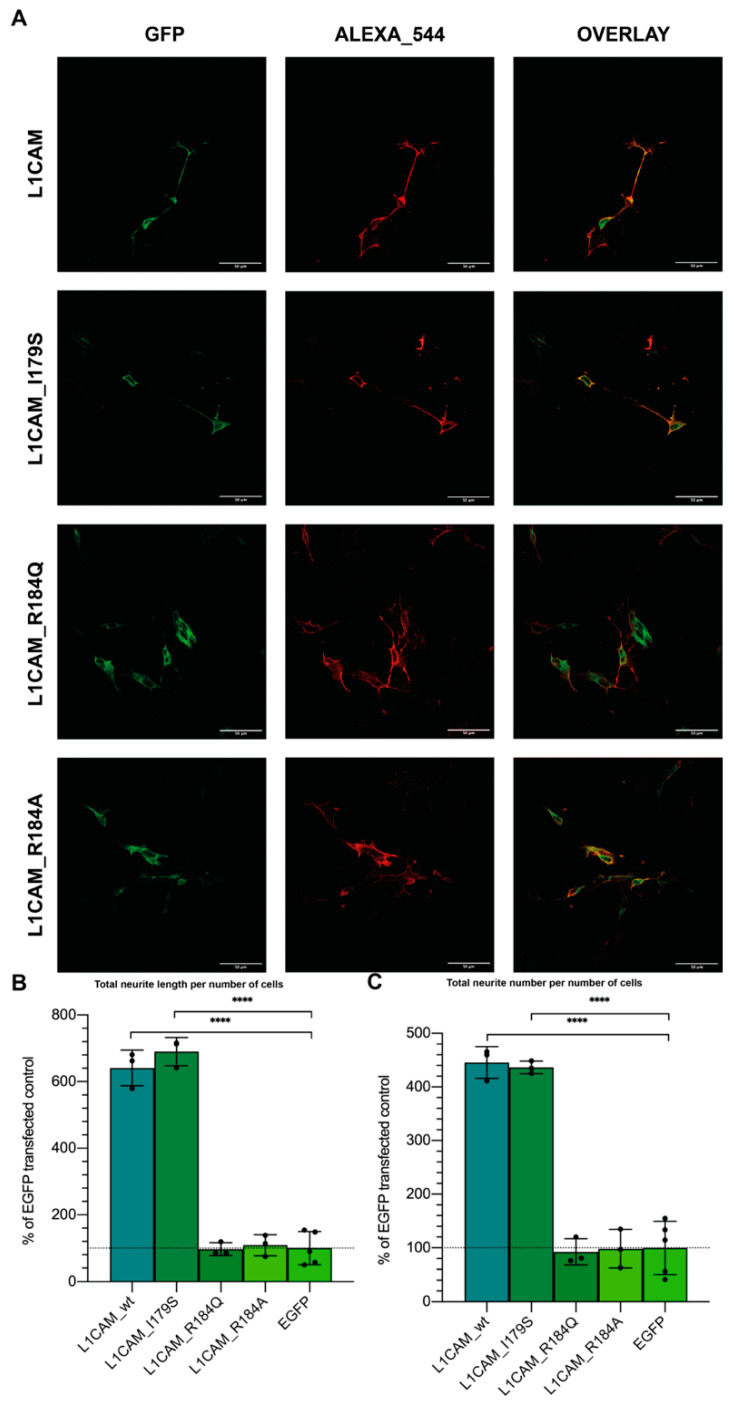
Neuritogenesis of proliferating SH-SY5Y cells transfected with L1CAM derived CDS. (**A**) Confocal images of GFP-tagged wt or mutant L1CAM-transfected cells. Treatment with secondary antibody was performed on nonpermeabilized cells. (**B**) Total neurite length per number of cells, (**C**) neurites per number of cells of proliferating SH-SY5Y cells transfected with indicated constructs. Neuritogenic potential is completely lost in transfection with L1CAM_R184A and L1CAM_R184Q, however is retained by L1CAM_I179S. Significance at *p* < 0.0001 (****) between samples and untreated control is reported.

The L1CAM I179S and R184Q mutations, responsible for CRASH syndrome, are located at the Ig2 binding site and the NOG motif subregion corresponding to the L1-A sequence. To set up an in vitro model for investigating the effects of the I179S, R184Q, and R184A mutations on L1CAM neuritogenic capacity, mutant versions of the L1CAM cDNA were obtained by mutagenesis. Proliferating SH-SY5Y cells were transiently transfected with wild type or mutant L1CAM_EGFP fusions, and their expression at the plasma membrane was confirmed by confocal microscopy (Figure 3A). Neuritogenic properties were compared, both in terms of total neurite length/number of transfected cells, and neurite number/number of transfected cells. As the basal expression of endogenous L1CAM in proliferating SH-SY5Y was neither suppressed by knockout nor by interference, transfection by EGFP alone was used for considering this baseline. 

Indeed, cells transfected with wild type L1CAM showed a tenfold increase in neuritogenesis, while those transfected with L1CAM_R184A did not show any improvement with respect to EGFP-transfected control (Figure 3B,C). Similarly, mutation L1CAM_R184Q, which is associated with a severe form of the CRASH syndrome, also strongly impaired neuritogenesis, which, instead, was only partially diminished by mutation I179S, in agreement with its association with a milder CRASH phenotype [45]. Intriguingly, in the regular expression representing relative conservation of residues in the NOG motif, R184 corresponds to position 7, where only Arg is accepted (100% conservation in an almost ten thousands CAMs dataset), whereas I179 corresponds to position 2, where both Ile and Ser are among accepted residues [22]. Overall, data confirm that when the Ig2 binding site is altered by mutation, the neuritogenic capacity of L1CAM can be diminished or even lost.

### 3.4. In Silico Simulations Suggest a Rationale for CRASH Differential Severity and Translational Perspectives

**Figure 4:**
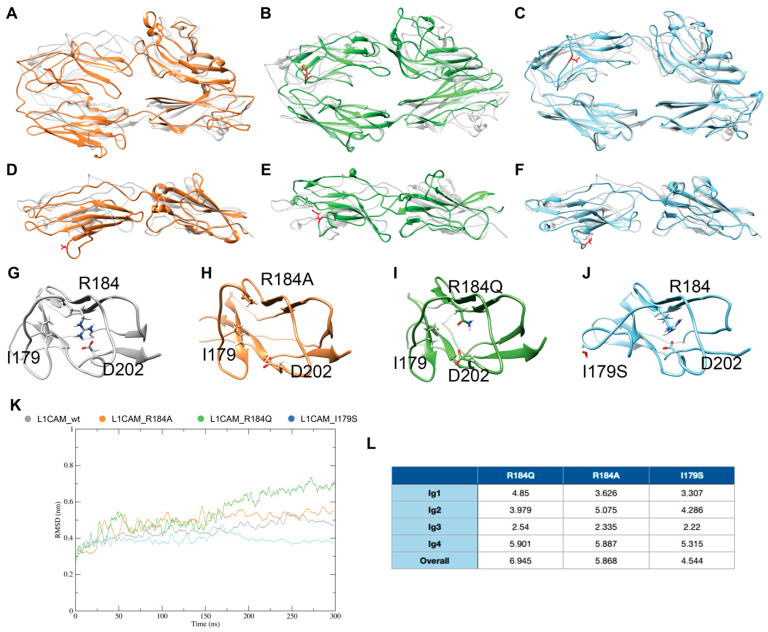
Molecular dynamics simulations of L1CAM and its mutants. Comparison of models after 300 ns simulations with wt (**A**–**C**) side view, (**D**–**F**) top view (orange: L1CAM_R184A, green: L1CAM_R184Q, light blue: L1CAM_I179S, white: L1CAM wild type). (**G**–**J**) Structural variation of Ig2 domains of WT, R184A, R184Q, and I179S mutants, respectively. (**K**) Variation of the root mean square deviation (RMSD) of the Ig2 domain during the simulation. (**L**) All-atom RMSD between mutants and wild type final structures. All data are reported in Å.

To gain insights into the molecular mechanisms underlying loss of function depending on the aforementioned CRASH mutations, the L1CAM horseshoe was modeled by homology using, as a template, the neurofascin homodimer crystal structure (3P3Y), which is the established structural prototype for proteins of the L1-family [6]. However, as both mutants and wild type L1CAM are modeled on such template, they might be biased towards it and relevant conformational differences might be masked. All models were thus driven toward a more informative “native” conformation by means of molecular dynamics simulation. Figure 4 shows that, consistently with the experimental data, L1CAM_R184Q and L1CAM_R184A undergo the most extensive conformational changes, whereas the L1CAM_I179S structure is similar to the wild type one. Such changes are particularly pronounced at the outer side of the Ig2 domain of the protein, which, according to its homology with Neurofascin dimer crystal structure, is thought to be involved in L1CAM homophilic interaction. Notably, when final structures are compared to the wild type one, all mutants show an overall all-atom root mean square deviation (i.e., the average distance between atoms of the two superposed proteins) above 4.5 Å. However, when considering alterations of the Ig2 fold, the R184A mutant undergoes the most extensive conformational changes (Ig2 RMSD compared to wt: 5.075), as the Arg to Ala mutation disrupts a salt bridge with Asp 202. Such salt bridge is also lost in the R184Q mutant. Even tough structural rearrangements caused by this mutation are less evident than in the R184A mutant (Ig2 RMSD compared to wt: 3.979), they are still enough to hamper L1CAM-mediated neurite outgrowth in in vitro assays. Seemingly, Ig2 with I179S mutation undergoes slightly higher conformational changes (Ig2 RMSD compared to wt: 4.286), but most of such variation is located in the 174-181 loop, and mutations of this region in neurofascin was found not to sensibly hamper homophilic binding in vitro [6].

**Figure 5:**
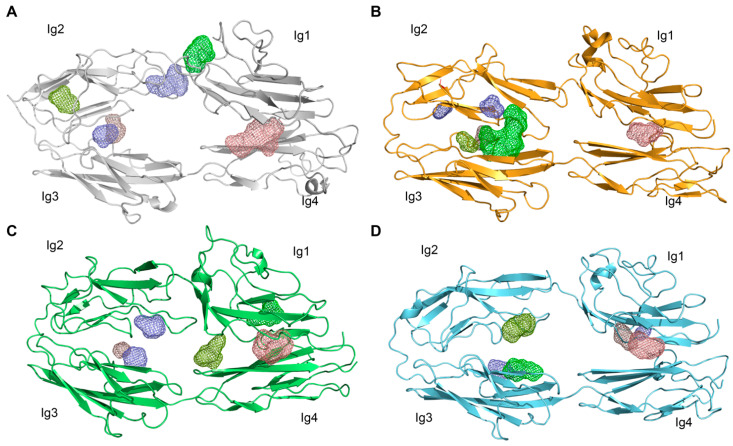
Prediction of putative peptide binding sites onto L1CAM horseshoe. (**A**) Wild type L1CAM, (**B**) L1CAM_R184A, (**C**) L1CAM_R184Q, (**D**) L1CAM_I179S. Each binding pocket is represented by a colored mesh.

To gain further insight about the possible peptide binding site on the L1 horseshoe structure, final models from MD simulations were then submitted to PeptiMap. As shown in Figure 5, all possible peptide binding sites were predicted on the inner side of the horseshoe, at either the Ig2–Ig3 or the Ig1–Ig4 interface; notably, such predicted binding pockets are retained by all mutants and, particularly, by the CRASH-causing mutant R184Q.

### 3.5. L1-A Can Restore Neuritogenesis on a CRASH Syndrome Cell Model, While L1CAM ED Is Unable

Preliminary in vitro and in silico evidence seems to confirm the neuritogenic capacity of L1CAM to strongly depend on Ig2-mediated binding, suggesting biomimetic peptides as tools to modulate L1CAM-induced neuritogenesis, and eventually for therapeutic purposes.

In addition to known usefulness of small biomimetic peptides as a biotechnological and biomedical tool, further advantages over L1CAM ED can be inferred by in silico evidence. Indeed, simulations suggested exogenous L1CAM ED might be unable to promote neuritogenesis in CRASH mutants because of conformational constraints. Instead, the much smaller L1-A and NOG peptides might overcome such limits and be able to complement CRASH-related neuritogenesis defects.

**Figure 6:**
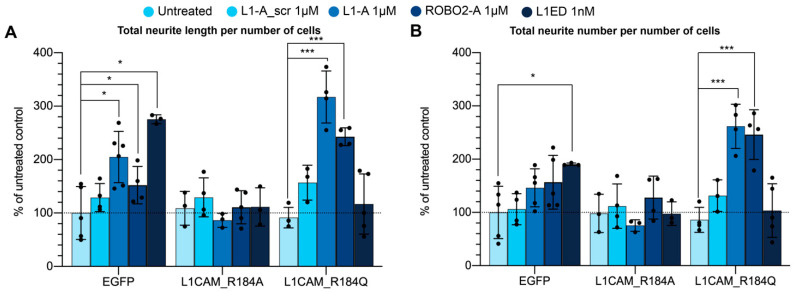
Rescue of CRASH cell phenotype by treatment with L1-A. (**A**) Total neurite length per number of cells and (**B**) neurite number per number of cells of proliferating SH-SY5Y cells transfected with indicated L1CAM mutants and treated with peptides. All data represent the mean ± SD of at least three independent experiments. Statistical analysis was performed using one way ANOVA with Tukey’s correction; significance at *p* < 0.05 (*), and *p* < 0.0001 (***) between samples is reported.

To suggest NOG-derived peptides as a possible therapy for forms of CRASH syndrome caused by mutations in the Ig2 domain, we used the previously described cell model. Particularly, cells transfected with L1CAM_R184Q (causing the most severe CRASH phenotype) were treated with either 1µM L1-A, 1nM L1CAM ED, 1 µM L1-A_scr [21], or left untreated, and compared with controls, i.e., EGFP-transfected cells. L1CAM_R184A was also included in the analysis as it is predicted to assume an even worse conformation than the naturally occurring mutation R184Q. As expected, EGFP transfection did not alter L1-A or L1CAM ED-mediated neuritogenesis (Figure 6). Coherently with in silico predictions, (i) the nonnatural mutation R184A could not be rescued by treatment with either L1CAM ED or L1-A, (ii) L1CAM ED was also ineffective with L1CAM_R184Q, while (iii) strikingly, L1-A and ROBO2-A could rescue such natural and severe mutation of L1CAM, strengthening the suggested therapeutic prospective.

## 4. Discussion

L1-CAM orchestrates the axon guidance during neurodevelopment and regeneration by mediating cell-cell adhesion through homo- or heterophilic interactions with other Ig superfamily members and integrins [3,4,5,46]. We previously identified a 14-aminoacids stretch from neuronal CAMs Ig-like domains that, when produced as synthetic peptides, can stimulate neurite outgrowth in neuron precursor-like cells [21,22,46]. Here, we compared the potency of such peptides and the entire L1CAM ED. We found that both NOG-derived peptides and L1CAM ED show comparable neuritogenic activity; dose-response curves have a sigmoidal shape and reach plateau around 1 µM and 1 nM, respectively. Once such plateau is reached, by treatment with either L1CAM ED or NOG peptides, neuritogenesis cannot be further improved by adding the other type of molecule. Moreover, treatment with L1-A_R184Q blocked L1CAM ED-mediated neuritogenesis. Overall, these data suggest an L1CAM ED biomimetic action for peptides. Although L1CAM ED requires a reduced dosage (1 nM) compared to the L1-A peptide (1 uM) to obtain the plateau of neuritogenic activity, the usage of synthetic peptides instead of recombinant or tissue-derived proteins offers multiple advantages such as low immunogenicity and reduced batch-to-batch variability, increased stability and easier functionalization of biomaterials, reduced production costs, and the possibility to be presented to cells at high surface densities [47]. Therefore, NOG-derived peptides hold great promises for developing neuroregeneration strategies.

Given the central role played by L1CAM in brain development, it is not surprising that mutations or polymorphisms of the *L1CAM* gene lead to severe neurological disorders summarized as CRASH syndrome. Specifically, some of the most severe cases are due to the R184Q mutation, while milder cases are due to the I179S mutation. 

Interestingly, both mutations are mapped within the NOG motif and the L1-A peptide. Our transfection of neuron precursor-like cells with WT and mutated L1CAM ectodomains reveals that only the WT and the I179S ectodomains support the neurite outgrowth, which is instead equal to the baseline in L1CAM_R184Q and L1CAM_R184A-transfected cells. Observations about the R184A and R184Q mutations are coherent with those of Zhao and co-workers [17]. Conversely, while human L1CAM_I179S mutation was not able to sustain neurite outgrowth in mice primary neurons [20], it is not different from L1CAM-transfected cells in our assay.

Consistent with these data, our incubation of neuron precursor-like cells with L1-A peptides bearing the R184Q, R184A, and I179S mutation shows that, when mutated at Arg-184, L1-A loses its neuritogenic properties, which are only partially impaired by mutation I179S, in line with the severity of the disease. Severity of mutations concerning R184 is, in turn, in agreement with the absolute conservation of corresponding arginine at position 7 of the NOG motif [17]. Taken together, these results suggest that R184 is a key amino acid conferring the L1CAM ED and the NOG-derived peptides the neurite elongation properties.

The neurite outgrowth is mediated by the homophilic binding occurring between two L1CAM Ig2 domains. Even though our molecular dynamics simulation reveals that the L1CAM_R184Q and L1CAM_R184A undergo extensive conformational changes in the Ig2 domain that could affect its ability to perform the homophilic binding, the PeptiMap analysis shows that the WT L1-A peptide retains the binding ability at the Ig interface in both the WT and mutant ectodomains, suggesting a possible bioactive effect.

As L1CAM mainly acts by homophilic binding, its expression onto the plasma membrane is required for peptide binding and triggering neurite outgrowth. In fibroblast-like COS-7 cells, membrane expression of L1CAM R184Q is controversial, as it has been found to be retained inside the endoplasmic reticulum, albeit with a considerable variability [48]. However, in the neuronal precursor-like cell line SH-SY5Y, our data indicate a considerable membrane expression of L1CAM_R184Q and L1CAM_R184A, suggesting that different cell types may apply different posttranslational controls modulating membrane expression of the same protein.

To confirm the possible bioactive effect of the WT peptide on mutant L1CAM ED, we compared the neuritogenic effect of L1CAM ED vs. NOG-derived peptide administration in neuron precursor-like cultures expressing mutant L1CAM ED. When incubated with the entire WT L1CAM ED, cultures expressing L1CAM_R184Q CRASH mutation cannot extend their neurites; conversely, the incubation with the WT L1-A peptide in the same mutant cultures rescues the neuritogenesis. Notably, L1CAM_R184A transfected cells treated with either NOG-peptides or L1CAM ED display a level of neuritogenesis comparable to control and lower than those transfected with EGFP. A similar effect is also observed when L1CAM_R184Q-transfected cells are treated with L1CAM ED. This observation suggests that misfolding of L1CAM Ig2 domain can hamper neuritogenesis; however, both mutant proteins are still able to bind NOG peptides and exogenous L1CAM ED. Conservation of trans-homophilic binding between mutants and exogenous L1CAM is not unexpected, as it has been proposed that such binding cooperatively involve the Ig1–4 (necessary to promote neuritogenesis), Ig5–Ig6, and FnIII-2 domains (stabilizing the interaction). On the other hand, conservation of peptide binding is supported by in silico prediction. Overall, data suggest the entire L1CAM ED is able to bind to both wild type and mutant L1CAM but cannot trigger neuritogenesis in R184A and R184Q mutants because of conformational constraints induced by Ig2 misfolding. Conversely, the smaller L1-A and ROBO2-A peptides can overcome such constraints and rescue the neurite outgrowth in the CRASH-causing mutant R184Q. Moreover, as ROBO2 is not part of the L1CAM family and it is not known to interact with L1CAM, it is likely that the shared effect of L1-A and ROBO2-A depends on a feature of the NOG motif itself rather than a specific property of the protein they are derived from.

In conclusion, here, we show that NOG-derived peptides can replace the L1CAM ED in stimulating neurite elongation in WT cultures and, contrarily to the L1CAM ED, they are able to boost the neuritogenesis in cultures expressing the L1CAM_R184Q CRASH mutation. Therefore, NOG peptides could find application not only in neural regeneration strategies to treat PNS and CNS injuries, but also in pre- and postnatal therapies addressing the correction of neurodevelopmental disorders such as the CRASH syndrome.
